# Importance and safety of autologous sperm cryopreservation for fertility preservation in young male patients with cancer

**DOI:** 10.1097/MD.0000000000019589

**Published:** 2020-04-10

**Authors:** Yinfeng Li, Jian Zhang, Hanfeng Zhang, Bo Liu, Guorong Wang, Maoqiu Cao, Bencui Fu, Hui Li, Qinghua Jiang, Lin Yu, Yang Xian, Bizhen Su, Xiaohui Jiang

**Affiliations:** a Sichuan Cancer Hospital & Institute, Sichuan Cancer Center, School of Medicine, University of Electronic Science and Technology of China; b Department of Human Sperm Bank, West China Second University Hospital of Sichuan University, Chengdu, Sichuan, China; Key Laboratory of Birth Defects and Related Diseases of Women and Children (Sichuan University), Ministry of Education, Chengdu, China.

**Keywords:** cancer, deoxyribonucleic acid, fertility preservation, sperm cryopreservation

## Abstract

With development of tumor treatment, survival time of patients with cancer is significantly prolonged. Therefore, the current emphasis is not only the survival, but also the quality of life, especially, it is crucial for young male cancer patients who are unmarried and maintaining fertility. However, the awareness of fertility preservation for these patients is currently insufficient.

To give physician and cancer patients more clear understanding of the importance and safety of sperm cryopreservation, so that achieve patient fertility benefits.

First, the knowledge level and attitudes about fertility preservation were investigated by surveying 332 cancer patients and 103 medical staff with questionnaires. Second, 30 male cancer patients (experimental group) and 30 normal donors (control group) were selected and their sperm samples were cryopreserved. The sperm quality was compared between cancer patients and normal donors, before and after antitumor treatment in the cancer patients, and before and after sperm cryopreservation in both groups.

In the questionnaire survey, we found that there were 70% to 80% of medical staffs and cancer patients lacked knowledge of fertility preservation, and 27.7% of patients worried that tumor and sperm cryopreservation might affect their offspring. In the sperm preservative experiment, we found that sperm quality in cancer patients was further damaged after radiotherapy/chemotherapy in addition to tumor itself had a negative effect. However, sperm deoxyribonucleic acid fragments were not affected by sperm cryopreservation although there were significant differences in sperm quality before and after sperm preservation in both groups.

Radiotherapy/chemotherapy would further damage sperm quality of young male cancer patients. Medical staff should be aware of importance of sperm cryopreservation for fertility preservation for these patients. It is also necessary that medical staff should inform the patient about the safety of sperm freezing and guide the patient to participate in sperm cryopreservation.

**Key Points**

What is already known about the topic?

- Globally, cancer has become second leading cause of death, the number of cancer-related death was reported to be 8.8 million in 2015. But the survival time of patients with cancer is significantly prolonged. The current emphasis is not only the survival, but also the quality of life, especially it is crucial for men who are unmarried, maintaining fertility. The damage to male fertility is not only by tumor itself, but also by the chemo-radiotherapy/targeted therapy, people's awareness of fertility preservation is insufficient. The main reason may be contributed to that both medical staffs and patients have no enough knowledge and correct attitude about fertility preservation.

What this paper adds

- The present study showed that sperm concentration and/or progressive sperm motility in tumor patients were significantly lower while sperm DNA fragments were significantly more in cancer patients than those in normal donors.
- Cryopreservation can reduce sperm motility, but did not influence the potential fertility by analyzing the semen parameters.
- There was no significant difference in DNA fragment index before and after freezing, suggesting that the freezing technique is safe and reliable and does not affect the patient's genetic function.

## 1 Introduction

Globally, cancer has become second leading cause of death, the number of cancer-related death was reported to be 8.8 million in 2015.^1^,^2^ In China, it was also reported that 10,000 patients were diagnosed as cancer every day.^[2]^ In terms of gender, the incidence of cancer in men is 61% higher than that in women, and the relative incidence index is 1.61 times.^2^,^3^ 15% of male patients are younger than 55 years old, and about a quarter of them are younger than 20 years old.^[4]^

However, with the development of the treatment level for tumor diseases, the survival time of patients with cancer is significantly prolonged. A report published in 2016 indicated that 5-year survival rate for people diagnosed with cancer were around 69%.^[2]^ Therefore, the current emphasis is not only the survival, but also the quality of life, especially it is crucial for men who are unmarried, maintaining fertility.

Ferlay et al reported that some tumor patients have azoospermia before treatment, indicating that the tumor itself might be one of the pathogenic factors of azoospermia.^[5]^ In addition, the damage of fertility caused by radiotherapy and chemotherapy is well known.^[5]^ This is because that testicular tissue is very sensitive to radiotherapy and some drugs such as alkylating agents and platinum clearly have a destructive effect on testicular tissue.^[6]^ Although damage to male fertility by tumor itself, and chemo-radiotherapy/targeted therapy is clear, people's awareness of fertility preservation (FP) is insufficient, especially in China.^7^,^8^,^9^ The main reason may be contributed to that both medical staffs and patients have no enough knowledge and correct attitude about FP.

During the antitumor treatment period, medical staff, especially nursing staff, should focus on the patient's long-term quality of life and health education. However, at the physician's level, many reports indicated that physicians have a lower response rate to FP. In a national survey, the response rates of physicians from United States, UK, and Japan were 15%, 37.6%, and 52%, respectively.^[10]^ For cancer patients, some patients concerned about the cost of sperm storage, and more patients worried about whether cryopreservation would affect their offspring and whether cryopreservation would have an impact on genetics or deoxyribonucleic acid (DNA).

Nowadays, there is an increasing topic about the FP and the safety of sperm cryopreservation. Epidemiological surveys have found that hereditary tumors account for less than 1% of all tumors,^[11]^ and a few families with hereditary tumors can achieve the desire to have healthy children through freezing techniques.^[12]^

Therefore, the purpose of the present study is to investigate the status of knowledge level and cognitive attitude of patients and medical staffs on FP and compare the sperm quality before and after radiotherapy/chemotherapy and before and after sperm cryotherapy. Thus, to make medical staff and patients have a clearer understanding of the importance and safety of sperm cryotherapy for FP in young male patients with cancer.

## 2 Materials and methods

### 2.1 Study population

First, according to another series of studies by our research team, we investigate the status of the knowledge level and attitudes on FP by surveying 332 cancer patients and 103 medical staff with questionnaires. Second, we selected 30 patients with malignant tumors who were diagnosed at the Sichuan Cancer Hospital from January 2018 to August 2018 as an experimental group. Thirty medical staffs and 30 cancer patients were also surveyed, the scores of the knowledge level, attitudes and behaviors were compared between medical staffs and cancer patients.

The selection of the patients was performed according to the following criteria:

(1) patients aged 16 to 45 years,
(2) patients with expected survival time ≥1 year and
(3) patients with expected completion of more than 3 radiotherapy program and/or chemotherapy cycles.

The average age of the 30 patients was 28 years, in which 20% of patients had child and 14% had been married. At the same time, we selected 30 age-, family status- and marriage-matched healthy men as a control group. The general information of the patients and the healthy controls was shown in Table 1 .

**Table 1:**
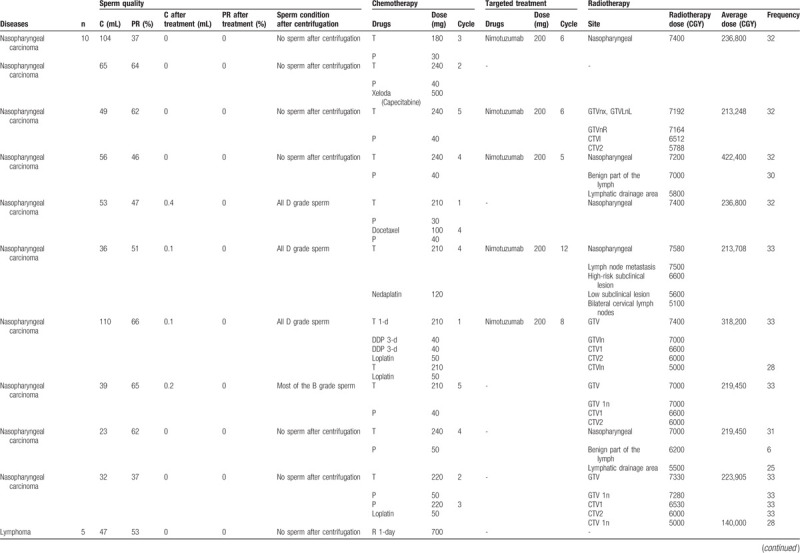
Tumor types and more information about the different cancer types in those 30 patients can be founded.

**Table 1 (Continued):**
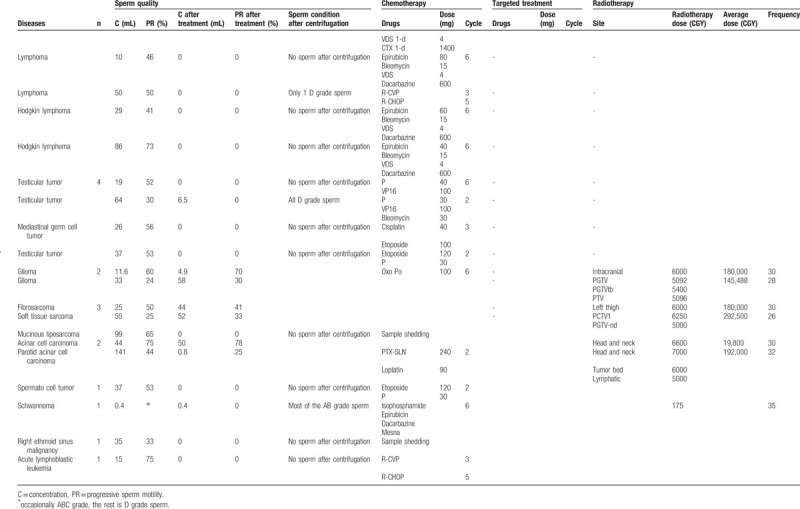
Tumor types and more information about the different cancer types in those 30 patients can be founded.

Physicians and cancer patients were respectively obliged to respond to 9 and 8 statements to demonstrate their knowledge of the effect of cancer treatments on fertility, or about FP. Each question with the right answer or a positive statement (ie, “I Know” or “Yes”) was scored as 1 point; otherwise, answers were scored as 0 point. As for FP attitudes, all response options to the items were on a 3-point Likert scale. One item was used to assess the degree of patient and physician's level of attention given FP (ie, not concerned to very concerned). Each option of each item was scored from 1 to 3, and the total possible overall attitude score was 15 points. Additionally, 8 items were used to evaluate practice behaviors on a 5-point Likert scale (never to always).

The study was approved by the Ethics Committee of Sichuan Cancer Hospital and all patients signed informed consent.

### 2.2 Autologous sperm preservation

First, we explained the process and precautions of the autologous sperm preservation to cancer patients and normal subjects. Second, the subjects signed the agreement and relevant information was registered into sperm bank system. Third, patient and normal subjects collected the semen by self-masturbation.

Afterward, we checked the sperm sample DNA fragmentation index by using sperm DNA fragmentation (SDF) staining Kit (Anhui Anke Biotechnology (Group) Co Ltd, China). Meanwhile, we performed the semen analysis including sperm concentration, progressive sperm motility (PR), mycoplasma culture, chlamydia culture, aerobic culture, and gonococcal culture by using Olympus CX41 phase contrast microscope, sperm counting board (Sefi-Medical Instrument, Israel), MyCoplasma IES Test kit (Zhuhai DL Biotech Co, Ltd, Guangdong, P.R. China), blood agar and chocolate agar.

Sterile CBS^TM^ high security tube (Cryo Bio System), sealer (Cryo Bio System SYMSIII) and cryoprotective solution (Self-prepared yolk-free modified Tyrode solution) were used for sperm freezing. The procedure of sperm freezing was as follow:

(1) the cryoprotectant and semen were added to the cryotube at a ratio of 1:3 and mixed, 1 mL per tube,
(2) the mixed semen samples were placed at 4°C for 15 minutes, and then placed 5 cm above the liquid nitrogen for 10 minutes, and
(3) placed in liquid nitrogen in an Isothermal Liquid Nitrogen Freezers (CBS, V1500AB).

When sperm were needed, the sample was taken out from the liquid nitrogen and placed at room temperature for 2 minutes, and then placed in a 37°C water bath for re-warming. The sample should be gently inverted and mixed, and then the sperm concentration, PR and SDF index after cryopreservation were analyzed.

### 2.3 Self-prepared yolk-free modified Tyrode solution

The self-prepared yolk-free modified Tyrode solution is a human sperm freezing protection solution containing no egg yolk and includes the following components and the final concentration of each component for sperm cryopreservation is: sodium chloride 90 to 110 mmol/L, Potassium chloride 5 to 6 mmol/L, magnesium sulfate 0.2 to 0.5 mmol/L, calcium chloride 2 to 4 mmol/L, sodium dihydrogen phosphate 0.2 to 0.5 mmol/L, sodium hydrogencarbonate 28 to 35 mmol/L, glycine 110 to 150 mmol/L, 4-hydroxyethylpiperazineethanesulfonic acid 18 to 25mmol/L, glucose 5 to 8 mmol/L, sucrose 30 to 60 mmol/L, sodium lactate 12 to 15 mmol/L, glycerol 50 to 100 mL/L, balance for ultra pure water. Since the solution does not contain egg yolk, it is not easy to cause an allergic reaction. The self-prepared yolk-free modified Tyrode solution is the patent we applied for (http://epub.sipo.gov.cn/patentoutline.action, Patent No: 2017103753849). It has a good protective effect on the freezing injury during sperm freezing and has no obvious negative impact on sperm physiological function, so it has good PR and recovery rate after sperm after resuscitation.

### 2.4 Evaluation

According to the requirements of the World Health Organization laboratory manual for the examination and processing of human semen (5th Edition),^[13]^ Sperm concentration, PR and DNA fragmentation index were 3 key indicators to evaluate the sperm quality. At least 200 sperms were calculated by Computer-aided sperm analysis (Beijing Suijia Software Co, Ltd. Beijing, P.R. China) to PR and repeated the detection twice. If the difference in test results is acceptable we will take the mean value or repeat the test twice if it is not acceptable.

SDF was defined as single or double strand breaks in nuclear DNA resulting in a potential loss/alteration of genetic information; sperm DNA integrity can be detected by sperm chromatin structure assay detects.^[14]^ Many genotoxic experiments showed excellent dose response data with very low coefficient of variation that further validated the sperm chromatin structure assay as being a highly powerful assay for sperm DNA integrity.^[14]^ The slide containing the semen sample was subjected to steps of dissolving, cooling, staining and rinsing, then researchers observed the results under a normal light microscope (40 × 10 × field of view), count at least 500 sperm and calculate the percentage of abnormal sperm.

Judging criteria for normal sperm and abnormal sperm: large halo and middle halo are normal sperm; small halo, no halo and degenerate sperm are abnormal sperm. Halo width/sperm head diameter ≥2/3 for large halo; 1/4 < halo width/sperm head diameter <2/3 for the halo ring; halo width/ sperm head diameter ≤1/4 for small halo Ring; no halo is observed as halo-free; spermatozoa in the sperm nuclei is degraded sperm.

### 2.5 Statistical methods

All analyses were conducted using SPSS Windows package (version 16.0, SPSS, Inc, Evanston, IL). Student *t* test was used compare the sperm concentrations and PR between normal controls and tumor patients. Paired sample *T* test and *Z* test were used to analyze the effects of antitumor therapy and sperm cryopreservation on sperm quality and sperm DNA fragments. A *P* < .05 was considered to be statistically significant.

## 3 Results

### 3.1 The trend of sperm quality and antitumor treatment

Table 1 illustrated the tumor types and more information about the different cancer types in those 30 patients can be founded.

### 3.2 Knowledge level and cognitive attitude of patients and medical staff on FP

According to another series of studies by our research team, as can be seen from a survey of 332 cancer patients,^[15]^ 77.8% of patients realized that tumors and their treatment would damage fertility, 27.7% of patients worried that tumors would affect the DNA of offspring, but 71.1% of patients still do not know the existence of sperm banks. Additionally, the knowledge of FP by medical staff was also limited. From the survey of 103 medical staff, 73.8% of medical staff did not consult the reproductive experts about fertility preservation, and 84.5% of medical staff did not even receive any knowledge of FP.

During the process of previous study, we compared the knowledge level, cognitive attitudes and practice behaviors of FP between 30 medical staffs and 30 cancer patients. The scores (mean ± standard deviation) of knowledge level and cognitive attitude on the patient's FP in medical staffs were 3.91 ± 1.67 and 12.29 ± 1.23, respectively. The scores in the patients were 3.50 ± 0.70 and 10.33 ± 0.95, respectively. These scores were relatively low when compared to full scores of knowledge level (8 points) and cognitive attitude (15 points). In addition, in the absence of knowledge and attitude, the practice of FP by medical staff and patients was 30.1% and 6.67%, respectively.

### 3.3 Effects of tumors and radiotherapy/chemotherapy on antitumor therapy on sperm quality

As shown in Table 2, the mean, SD and median of sperm concentration in patients with tumor before antitumor treatment were lower than those in normal subjects (*P* = .010). Compared to normal donors, the DNA fragments of tumor patients were significantly more (*P* = .0001).

**Table 2:**
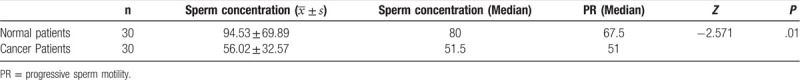
Comparison of semen analysis between cancer patients and normal sperm donors.

After anti-tumor treatment, 25 cases of the 30 patients continued to perform sperm quality testing. No sperm was detected after centrifugation in 11 cases, and D-class sperm was detected in 6 cases. Additionally, the sperm concentration and PR of the tumor patients after treatment were lower than those of the tumor patients before treatment (all *P* = .001) (Table 3).

**Table 3:**
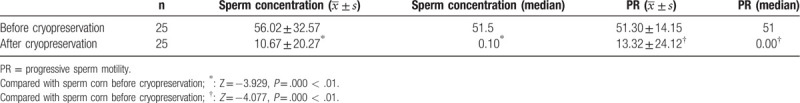
Comparison of sperm analysis before cryopreservation and after antitumor treatment in cancer patients.

### 3.4 The effect of sperm cryopreservation on sperm quality and DNA fragment integrity

The sperm concentration and PR after the sperm cryopreservation were significantly lower than that before cryopreservation in both cancer patient and normal donors (all *P* = .0001, Table 4).

**Table 4:**
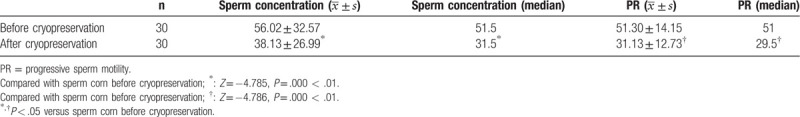
Comparison of sperm quality before and after cryopreservation in patients with cancer.

Compared with the DNA fragments of spermatozoa before cryopreservation, the DNA fragments after cryopreservation were slightly more but did not reach statistically significant in both groups (*P* = .829) (Table 5).

**Table 5:**
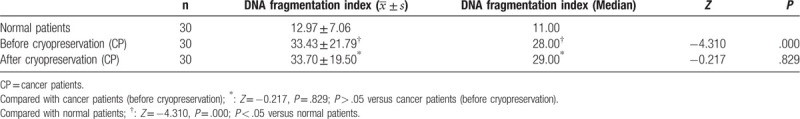
Comparison of DNA fragment index (%) among normal patients, tumor patients before and after cryopreservation.

## 4 Discussion

The impact of tumor itself on sperm quality has been well documented.^[16]^ Auger et al demonstrated that various types of tumors can have a series of effects on sperm quality in young tumor patients when compared the sperm quality between 4480 young cancer patients and 1148 healthy sperm donors.^[17]^ Williams et al showed that testicular tumors may reduce the sperm quality by directly damaging germ cells^[18]^ and van Casteren et al revealed that other types of malignant tumors such as leukemia, lymphoma, and gastrointestinal tract increase the risk of azoospermia in men.^[19]^ In addition, some patients with tumors have had azoospermia before treatment, which fully indicates that the tumor itself is one of the pathogenic factors of azoospermia.^[5]^ The present study showed that sperm concentration and/or PR in tumor patients were significantly lower while sperm DNA fragments were significantly more in cancer patients than those in normal donors, that are consistent with the findings of other studies described above. This study was also help newly diagnosed young male cancer patients make important decisions such as to pursue cryopreservation before undergoing treatment.

Here, the most important point we should emphasized is that antitumor treatment such as radiotherapy and/or chemotherapy would further damage the sperm quality in these patients.^[5]^ This is because that testicular tissue is extremely sensitive to radiotherapy and has a clear dose-related, and most chemotherapeutic drugs have reproductive toxicity.^[20]^ In addition, other treatment options for tumors such as surgery and targeted therapy could also lead to a decline in male fertility.^[21]^

In the present study, we also showed that azoospermia or low-class sperm occurred in more than 60% of tumor patients after antitumor therapies. Therefore, autologous sperm cryopreservation is necessary before treatment for the patients who wish to preserve their fertility.

Although sperm quality is damaged by tumor itself, it is believed that cryopreservative sperm from cancer patients before treatment can be used in In Vitro Fertilization- Intracytoplasmic Sperm Injection programs.^[22]^ A number of success cases to father their genetic children have been reported.^[23]^ In addition, it is worth mentioning that the emergence of (intra cytoplasmic sperm injection) technology allows a small number of viable sperm to complete the conception process. This greatly reduces the need for sperm quality and indicates the importance of semen preservation, enabling more patients to obtain fertility opportunities from autologous semen preservation. However, it needs to follow-up to observe whether the cryopreservative sperm of the 30 cancer patients in the present study can create babies in the future studies.

Concerning effect of cryopreservation on sperm quality, it has been reported that cryopreservation can reduce sperm motility, but did not influence the potential fertility by analyzing the semen parameters in 14 years of cryopreservative semen samples^[19]^ and some researchers believe that long-term cryopreservation had better safety, effectiveness and recovery rate.^[24]^ Furthermore, according to the World Health Organization laboratory manual for the examination and processing of human semen, the lower limit of the reference value of sperm concentration is 12 to 16, and the lower limit of reference value of PR is 31 to 34.^[25]^ In the present study, the mean of sperm concentration and PR after freezing were 38.13 and 31.13, respectively that are close to the reference values, which may be enough to satisfy artificial fertility^[25]^ although sperm quality was reduced after cryopreservation.

DNA fragment index is an indicator for sperm integrate and a novel marker for sperm fertility.^[26]^ Some studies pointed out that the reasons for parents to give up sperm freezing include the safety of pregnancy, or the genetic risk of malignant tumors.^[27]^ However, in the present study, we showed that there was no significant difference in DNA fragment index before and after freezing, suggesting that the freezing technique is safe and reliable and does not affect the patient's genetic function.

In addition to a clear genetic syndrome, there is no evidence that tumor itself, anti-tumor treatment or fertility intervention may increase the risk of cancer or congenital malformations in future generations.^[1]^

China's fertility protection technology is carried out far later than developed countries. Although sperm freezing is a reproductive benefit for cancer patients, especially unmarried young men, only small number of participants join the program. At present, there is a lack of research on fertility protection provided by oncologists in China, and the lack of information on sperm freezing in cancer patients is still a common phenomenon in society.

In conclusion, the present study demonstrated that tumor treatment can further damage the sperm quality in addition to tumor itself. Therefore, sperm freezing is very necessary in young cancer patients, and the freezing time is best before anti-tumor treatment. We also demonstrated that the cryopreservation did not significantly affect the sperm integrate and fertility potential. However, the majority of physicians and cancer patients lack the knowledge and the correct attitude about sperm cryopreservation for male FP. The National Comprehensive Cancer Network Guidelines for Adolescents and Young Patients with Malignant Tumors suggest that physicians should communicate treatment-related reproductive toxicity and fertility protection measures with patients and their families before treatment, and recommend reproductive specialists for patients with fertility needs.^[27]^ Therefore, it is the responsibility for physician to advise the tumor patient to perform sperm cryopreservation before treatment, and to inform the patient of the benefits of cryopreservation, to eliminate the patient's concerns that sperm freezing may affect the DNA of his offspring, and to achieve reproductive benefit for young male patients.

## 5 Limitation and future implications

The sample size of this study was small and included different types of tumor. The sperm analysis of 5 patients was not collected after anti-tumor treatment. However, as this study is a continuous research, follow-up work on patients after treatment is ongoing, and sperm analysis after treatment is still collecting. Future research can expand the sample size, focus on the single disease, and analyze the relationship between different diseases in male sperm quality and fertility. In addition, future research should also focus on DNA damage and chromosomal aberrations, embryonic development abnormalities, and birth defects.

## Acknowledgment

The authors would like to thank the staff in the department of human sperm bank, who helped in collecting data. Many thanks to Professor Guorong Wang for her advice on data statistics and the handling of SPSS. We also would like to thank the Scientific Research of Sichuan Province Health Department for the financial support.

## Author contributions

**Conceptualization:** Yinfeng Li, Jian Zhang, Hanfeng Zhang, Lin Yu, Xiaohui Jiang.

**Data curation:** Yinfeng Li, Bencui Fu, Yang Xian, Bizhen Su.

**Formal analysis:** Yinfeng Li, Maoqiu Cao, Hui Li.

**Funding acquisition:** Jian Zhang.

**Methodology:** Hanfeng Zhang.

**Project administration:** Bo Liu, Guorong Wang, Maoqiu Cao, Xiaohui Jiang.

**Resources:** Bo Liu, Bencui Fu, Lin Yu.

**Software:** Guorong Wang.

**Supervision:** Hanfeng Zhang, Guorong Wang, Maoqiu Cao, Qinghua Jiang, Lin Yu, Yang Xian, Bizhen Su.

**Validation:** Bencui Fu, Qinghua Jiang.

**Visualization:** Hui Li, Qinghua Jiang.

**Writing – original draft:** Yinfeng Li.

**Writing – review and editing:** Yinfeng Li, Xiaohui Jiang.

## References

[1] Byrne J Rasmussen SC Steinhorn RR Genetic disease in offspring of long-term survivors of childhood and adolescent cancer. Am J of Hum Genet 1998;62:45–52. 9443870 10.1086/301677 PMC1376803

[2] WHO. (2018). Available at: http://www.who.int/mediacentre/factsheets/fs297/en/. Accessed 16 August 1998.

[3] Polland A Berookhim BM Fertility concerns in men with genitourinary malignancies: treatment dilemmas, fertility options, and medicolegal considerations. Urol Oncol 2016;34:399–406. 27283219 10.1016/j.urolonc.2016.05.007

[4] Heyu P Rongfshou Z Xibing S Analysis of gender differences in malignant tumors in China. J China Cancer 2013;22:174–9.

[5] Ferlay J Steliarova-Foucher E Lortet-Tieulent J Cancer incidence and mortality patterns in Europe: estimates for 40 countries in 2012. Eur J Cancer 2013;49:1374–403. 23485231 10.1016/j.ejca.2012.12.027

[6] Xiaohua L Jiabao W Yunge T Effect of tumor and its treatment on fertility of male patients and genetic risk analysis. Chin J Fam Plan 2018;26:415–9.

[7] Trost LW Brannigan RE Oncofertility and the male cancer patient. Curr Treat Options Oncol 2012;13:146–60. 22528369 10.1007/s11864-012-0191-7

[8] Hallak J Mahran AM Agarwal A Characteristics of cryopreserved semen from men with lymphoma. J Assisted Reprod Genet 2000;17:591–5. 10.1023/A:1026443510493 PMC3455457 11209541

[9] Centola GM Keller JW Henzler M Effect of low-dose testicular irradiation on sperm count and fertility in patients with testicular seminoma. J Androl 1994;15:608–13. 7721664

[10] Ronquist G Brody I The prostasome: its secretion and function in man. Biochim Biophys Acta 1985;822:203–18. 2992593 10.1016/0304-4157(85)90008-5

[11] Pacey AA Merrick H Arden-Close E Monitoring fertility (semen analysis) by cancer survivors who banked sperm prior to cancer treatment. Hum Reprod 2012;27:3132–9. 22926842 10.1093/humrep/des300

[12] Burrell RA McGranahan N Bartek J The causes and consequences of genetic heterogeneity in cancer evolution. Nature 2013;501:338–45. 24048066 10.1038/nature12625

[13] Xingwu C Kai L Cuiying L A review of WHO laboratory manual for the examination and processing of human semen(5th edition). National Journal of Andrology 2011; 17: 1059–1063. 22235670

[14] Donald PE The sperm chromatin structure assay (SCSA^®^) and other sperm DNA fragmentation tests for evaluation of sperm nuclear DNA integrity as related to fertility. Anim Reprod Sci 2016;169:56–75. 26919909 10.1016/j.anireprosci.2016.01.017

[15] Zhang H Wang G Jiang B The knowledge, attitude, and self-reported behaviors of oncology physicians regarding fertility preservation in adult cancer patients. J Cancer Educ 2019;doi: 10.1007/s13187-019-01567-6. 10.1007/s13187-019-01567-6 PMC7679324 31256354

[16] de Pedro M Otero B Martin B Fertility preservation and breast cancer: a review. Ecancermedicalscience 2015;9:503–23. 25729416 10.3332/ecancer.2015.503 PMC4335963

[17] Auger J Sermondade N Eustache F Semen quality of 4480 young cancer and systemic disease patients: baseline data and clinical considerations. Basic Clin Androl 2016;26:3–13. 26893905 10.1186/s12610-016-0031-x PMC4758099

[18] Williams DH IV Karpman E Sander JC Pretreatment semen parameters in men with cancer. J Urol 2009;181:736–40. 19091343 10.1016/j.juro.2008.10.023

[19] van Casteren NJ Boellaard WP Romijn JC Gonadal dysfunction in male cancer patients before cytotoxic treatment. Int J Androl 2010;33:73–9. 19538481 10.1111/j.1365-2605.2009.00956.x

[20] Wyrobek AJ Relative susceptibilities of male germ cells to genetic defects induced by cancer chemotherapies. J Natl Cancer Inst Monogr 2005;34:31–5. 10.1093/jncimonographs/lgi001 15784819

[21] Smith GD Serafini PC Fioravanti J Prospective randomized comparison of human oocyte cryopreservation with slow-rate freezing or vitrification. Fertil Steril 2010;94:2088–95. 20171613 10.1016/j.fertnstert.2009.12.065

[22] Sheng HQ Zhang XZ Hong Y Analysis of the quality of cryopreserved semen from male cancer patients. Zhonghua Nan Ke Xue 2015;21:44–7. 25707139

[23] Katz DJ Kolon TF Feldman DR Fertility preservation strategies for male patients with cancer. Nat Rev Urol 2013;10:463–72. 23835578 10.1038/nrurol.2013.145

[24] Yogev L Kleiman SE Shabtai E Long-term cryostorage of sperm in a human sperm bank does not damage progressive motility concentration. Hum Reprod 2010;25:1097–103. 20176594 10.1093/humrep/deq041

[25] Ståhl O Boyd HA Giwercman A Risk of birth abnormalities in the offspring of men with a history of cancer: a cohort study using Danish and Swedish national registries. J Natl Cancer Inst 2011;103:398–406. 21303994 10.1093/jnci/djq550 PMC3046951

[26] Kort JD Eisenberg ML Millheiser LS Fertility issues in cancer survivorship. CA Cancer J Clin 2014;64:118–34. 24604743 10.3322/caac.21205

[27] Lawson AK Klock SC Pavone ME Psychological counseling of female fertility preservation patients. J Psychosoc Oncol 2015;33:333–53. 25996581 10.1080/07347332.2015.1045677 PMC4557874

